# Temperature Sensing and Honey Bee Colony Strength

**DOI:** 10.1093/jee/toac034

**Published:** 2022-05-06

**Authors:** Daniel Cook, Boyd Tarlinton, James M McGree, Alethea Blackler, Caroline Hauxwell

**Affiliations:** School of Biology and Environmental Science, Faculty of Science, Queensland University of Technology, 2 George St, Brisbane City, QLD 4000, Australia; School of Biology and Environmental Science, Faculty of Science, Queensland University of Technology, 2 George St, Brisbane City, QLD 4000, Australia; School of Mathematical Sciences, Faculty of Science, Queensland University of Technology, 2 George St, Brisbane City, QLD 4000, Australia; School of Design, Faculty of Creative Industries, Education and Social Justice, Queensland University of Technology, 2 George St, Brisbane City, QLD 4000, Australia; School of Biology and Environmental Science, Faculty of Science, Queensland University of Technology, 2 George St, Brisbane City, QLD 4000, Australia

**Keywords:** *Apis mellifera*, honey bee, pollination, temperature, strength

## Abstract

Strength auditing of European honey bee (*Apis mellifera* Linnaeus, 1758 [Hymenoptera: Apidae]) colonies is critical for apiarists to manage colony health and meet pollination contracts conditions. Colony strength assessments used during pollination servicing in Australia typically use a frame-top cluster-count (Number of Frames) inspection. Sensing technology has potential to improve auditing processes, and commercial temperature sensors are widely available. We evaluate the use and placement of temperature sensing technology in colony strength assessment and identify key parameters linking temperature to colony strength. Custom-built temperature sensors measured hive temperature across the top of hive brood boxes. A linear mixed-effect model including harmonic sine and cosine curves representing diurnal temperature fluctuations in hives was used to compare Number of Frames with temperature sensor data. There was a significant effect of presence of bees on hive temperature and range: hives without bees recorded a 5.5°C lower mean temperature and greater temperature ranges than hives containing live bees. Hives without bees reach peak temperature earlier than hives with bees, regardless of colony strength. Sensor placement across the width of the hive was identified as an important factor when linking sensor data with colony strength. Data from sensors nearest to the hive geometric center were found to be more closely linked to colony strength. Furthermore, a one unit increase in Number of Frames was significantly associated with a mean temperature increase of 0.36°C. This demonstrates that statistical models that account for diurnal temperature patterns could be used to predict colony strength from temperature sensor data.

Many flowering crops such as almonds, avocado, and blueberry are grown in large scale monocultures which exclude native pollinator populations through habitat removal and scale of planting (Kearns et al. 1998, Breeze et al. 2011). To rectify this, managed hives of European honeybees *Apis mellifera* L., are transported to orchards to pollinate the crop for the flowering period, with the global value of pollination services estimated at AUD240 billion, and increasing (Potts et al. 2010, Workman 2016). Colony-strength auditing is a key proxy measure for pollination efficacy (Sheesely and Poduska 1970), and is the critical assessment for bee-brokers to deliver pollination success and equitable payment in contract pollination services, particularly in almonds.

The supply of weak hives is a substantial factor in poor pollination events, with effects ranging from low flower visitation (affecting fruit set) to low pollination radius. In some cases, the risk of disease transmission from weak hives during hive musters may deter apiarists from providing pollination services (Sheesely and Poduska 1970, Goodman 2015). Detection of weak hives is also of critical importance in apiaries, where pests and diseases may be spread by stronger colonies robbing stores from weaker, infected hives (Genersch 2008, Goodman 2015).

The terms “colony health” and “strength” are often interrelated and require some definition; “colony health” includes multiple variables, including queen-rightness, open and capped brood, “disease” levels (viral, fungal, and insect pest) and nectar and pollen stores (Goodman 2015). “Colony strength” refers to population size within a colony, particularly workers able to forage for nectar or pollen (typically aged 20+ d) and should also reflect the number of future worker bees in the form of open and capped brood (Seeley 1982, Nasr et al. 1990, Delaplane et al. 2013). Rigorous, objective, and empirical methods to determine colony strength include measurements such as total bee weight (Kg), brood area (cm^2^), brood solidness (percentage of capped brood cells), honey weight, and disease presence and prevalence (Delaplane et al. 2013).

A less disruptive but more subjective method of colony assessment uses a pair of observers to remove frames from the hive and visually estimate bee coverage as a percentage of a frame side. The auditor may then calculate total bees per frame using the frames’ comb area and multiplying by 1.23–1.77 bees per cm^2^ (Delaplane et al. 2013). Within Australian apiculture, a more common metric is to count the number of “Frames of Bees” (FOB) that are 75% or more covered with bees at 15°C (Somerville and Frost 2018).

This Frames of Bees method has been used to directly correlate colony strength to pollination services through pollen foraging and weight of pollen collected (Sheesley and Poduska 1970), and typical hive standards specified in pollination contracts require hives supplied to be queen-right, disease free and contain 8 or more frames of bees with 75% coverage of bees at 15°C (Somerville and Frost 2018). However, this is rarely used in practice due to constraints on the cost-effective and timely audit of tens of thousands of hives, and the impacts of colony disruption and cool weather during frame-by-frame audits (Delaplane et al. 2013).

In practice, a less invasive “cluster count method” is most often used for in-orchard audits, where strength is determined from frame-tops covered in bees i.e. Number of Frames (Nasr et al. 1990, Somerville and Frost 2018). This requires only inspection of the tops of the frames of each hive box and is thus less invasive and quicker than frame by frame inspections but is also more subjective.

This cluster count method is accurate for colonies around the 8-frame range commonly stipulated in pollination service contracts but may overestimate the size of larger colonies (where Number of Frames >9 frames, often across 2 boxes), and underestimate the size of small colonies (where Number of Frames <3 frames) (Nasr et al. 1990).

The conversion between Number of Frames and Frames of Bees is not one-to-one. Equation 1 describes the conversion between the cluster count audit result “Number of Frames” to the frame-by-frame audit result in “Frames of Bees” (Nasr et al. 1990).


$$\begin{array}{l} Frames\;of\;Bees\;=\;2.114\;+\;(0.637\cdot Number\;of\;Frames) \end{array}$$ (1)

There are further limits on the effective and accurate delivery of hive audits even using this simplified cluster count method. The method is only effective within a narrow band of times (early morning) and temperatures (<=15°C) due to dispersal of clusters and increased forager output as the day progresses (Sheesley and Poduska 1970, Nasr et al. 1990). Furthermore, the practical difficulties of auditing tens of thousands of hives in orchards in a short time window requires very rapid assessment (a few seconds per hive) of just 10% of hives delivered and relies heavily on the tacit knowledge of the auditors (Phillips 2014, Somerville and Frost 2018).

The high value of apiculture services and advances in sensing technology have created a thriving area of innovation in hive sensing technology for use in both apiary and auditing termed “precision beekeeping” (Foth et al. 2016). Precision beekeeping technology is increasingly posited for use in assessment of colony strength as an alternative to conventional auditing processes (Edwards-Murphy et al. 2016, Zacepins et al. 2016). In practice, applications of sensing technology in both apiary management and auditing requires further detailed work to link sensing data accurately to colony strength with the confidence required to support both investment by the apiarist and to meet the needs of contracted pollination services.

Temperature is a critical factor in colony health and is actively managed by *A. mellifera* using heating and cooling behaviors to maintain a stable nest temperature close to the ideal of 35°C, and within the range 32–36°C (Tautz 2008, Stabentheiner et al. 2010). The maintenance of “hive homeostasis” is a good indicator of colony health, state, and even pupation activity at a comb-cell level (Tautz et al. 2003, Becher and Moritz 2009, Meikle et al. 2015, 2017, Abou-Shaara et al. 2017).

In experimental work, temperature sensing has shown associations between colony health and strength (Stalidzans and Berzonis 2013, Kridi et al. 2016, Meikle et al. 2017, Melicher et al. 2019). Temperature sensing using analysis of thermal imaging is now in commercial use (The Bee Corp 2021) and several commercial products include temperature sensing (Arnia.co.uk, Beehero.io, Broodminder.com, Smartbeekeeper.com).

Temperature sensing technology in some commercial applications uses digital sensors placed at either single or multiple points within the hive (Becher and Moritz 2009, Edwards-Murphy et al. 2015a, b, 2016, Gil-Lebrero et al. 2016, Jiang et al. 2016, Kridi et al. 2016, Zacepins et al. 2016, Stalidzans et al. 2017). The placement of single sensors is typically in the geometric center of the hive rather than, for example, directly over the brood nest center (Stalidzans and Berzonis 2013, Zacepins et al. 2016, Meikle et al. 2015, 2017). However, heat distribution within a hive is not uniform. Hive construction and insulation affect changes in internal temperature relative to ambient, air movements affect heat distribution, honey and other stores act as thermal mass within the hive, and the bees themselves modify the environment through location of brood and thermal regulation (Cook et al. 2021a, Humphrey and Dykes 2008, Tautz 2008, Mitchell 2016).

This paper addresses key knowledge gaps in the use of auditing standards and temperature sensing to determine “hive strength”. The experiment was conducted in environmental conditions and time frame similar to those found in preparation for Australian almond pollination events, and while targeted to this event, the research and model may have relevance in colony strength measurement in other scenarios. The presented model analyses the relationship between temperature data, presence of bees, and colony strength measured by cluster count method (Nasr et al. 1990) in Number of Frames, and describes the effect of sensor placement in the hive.

## Materials and Methods

### Hives and Hive Audits

Forty-two (*n* = 42) single 10-frame, full depth, Langstroth wooden hives each containing 9 frames (as commonly used in Australian industry [honeybeesuite.com 2010, Beekeepingmadesimple.com 2020]) containing built-out comb were used. Each hive was fitted with a standard deep (50mm) migratory-style ventilated lid and a commercial “Hive Doctor” ventilated base (Ecrotek Ltd, New Zealand). A solid-bottomed hive baseboard was placed on top of each hive to reduce the impact of hive warming due to solar radiation. Hives were located on hive stands approximately 400 mm off the ground. Each hive stand had six hives, separated by ~150 mm, and is referred to as a group. The groups were set in two rows, one row of four groups and one row of three groups. Each group was separated from its row neighbors by ~2 meters, with ~5 meters between the two rows.

Forty (*n* = 40) hives were verified as disease free, with having an active laying queen, eggs, open and closed brood of varying amounts, nectar, capped honey, and pollen stores. Two additional (*n* = 2) hives without an active bee colony but containing 9 frames of empty wax comb were used as a control.

Colony strength audits were conducted as described in the Australian state government guidebook “AgGuide: Pollination using honey bees” using the “cluster count” top-of-frame assessment of “Number of Frames” (Somerville and Frost 2018). The audits and experiment were conducted in early winter (late May 2020 in Queensland, Australia). It should be noted that the apiary location in Queensland, Australia (–27.38773, 152.87827) is subtropical, and colonies do not, therefore, display winter clustering behaviors seen in cooler temperate zones. Audits were conducted on three nonconsecutive days prior to the start of the experiment (10^th^ May 2021), in random hive order, at temperatures <15°C, and starting at 06:00 hrs with photographic validation of Number of Frames as described below.

**Fig. 1.**
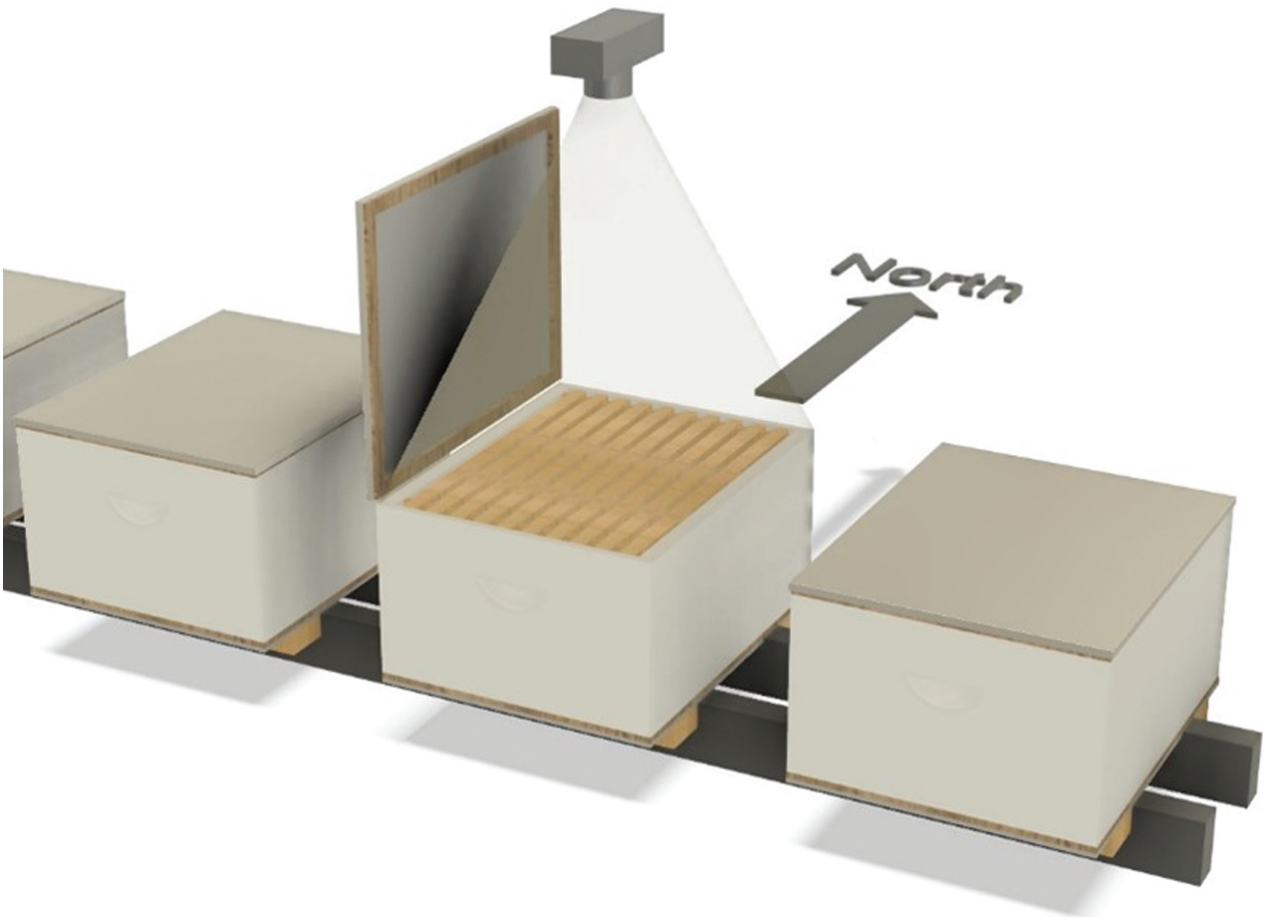
Number of Frames audit of the beehive used photographic capture of the frame top prior to assessment of the photographic image by two auditors. Hive lids were opened on the west side of north facing hives.

Audits were performed by first breaking the propolis seal with a hive tool then opening the lid to 90° from horizontal on the west edge of the north facing hive (Fig. 1). The frame tops were then photographed on a Samsung Galaxy A71 Phone and the lid replaced. No assessments were made in the field. The photographs were then compared to a reference grading scale generated from industry training literature (Somerville and Frost 2018) to create a Number of Frames strength score in photographic assessments by two independent assessors. One assessor was experienced in the cluster count methodology used in the almond industry, and one assessor was a novice at the cluster count method. Disparity between assessments >=2 Number of Frames was reassessed by both auditors independently.

A test for Pearson’s product-moment correlation was used to assess whether the audits by the two independent assessors were correlated. A mean of each auditor’s assessment at each 06:00 am timepoint was calculated and used to produce a mean Number of Frames with standard deviation for each hive.

### Sensors

**Fig. 2.**
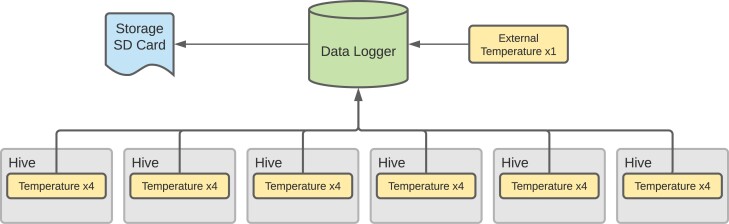
Connection diagram of sensor arrays to data logger unit. Six temperature arrays containing four temperature sensors were in each of the six hives in a group, and a single temperature sensor measured external ambient temperature. Data was logged to a MicroSD data storage card which was removed and read at the end of the experimental period.

The 42 hives were equipped with sensor systems. Each hive contained four temperature sensors. Hives in groups of six on a single stand were connected to a master unit logging data from the four sensors in each of the six hives and an external ambient temperature sensor (*n* = 25 sensors per group, with 7 groups) (Fig. 2).

Custom designed temperature loggers were created using Maxim Integrated DS18B20 1-Wire Digital Thermometer (Maxim Integrated 2021) with a ±0.5°C accuracy over –10°C to 85°C, 0.0625°C resolution (12 bit), running in powered (nonparasitic) mode, and factory calibrated.

**Fig. 3.**
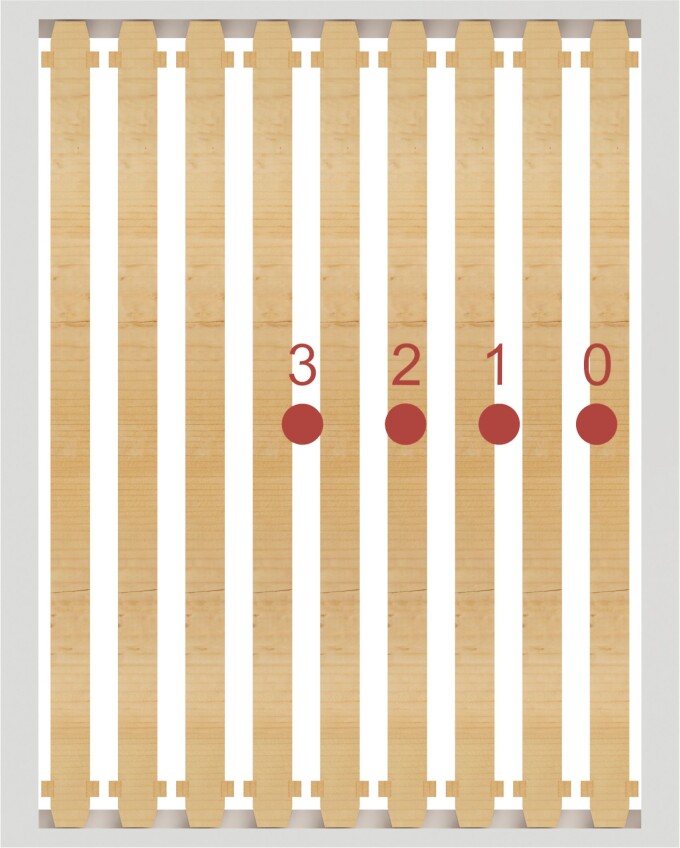
Sensor position across a North facing (upward) Langstroth hive containing nine frames. Sensor numbers run sequentially from outside to center.

A custom-designed printed circuit board containing an array of four DS18B20 sensors spaced at 60 mm intervals (Fig. 3) was used to record temperature over time from the center of the hive to the outside edge of the hive, and each hive was equipped with this sensor array circuit board.

Observations were recorded as records of unique 16-bit sensor serial number, time zone adjusted Unix date-time stamp, and temperature measurement.

### Data Collection

Data collection intervals and duration of experiment used a method modified from Meikel et al. (2015). Data were recorded at 5-min intervals for 18.6 d commencing on the 10^th^ May 2021. At the end of the experiment, microSD data cards were collected, read and the data were formatted in Microsoft Excel, version 2105, then transferred to R version 4.1.0 with R Studio, version 1.4.1103, which was used for all downstream analysis (Microsoft Corporation 2018, RStudio Team 2020).

Date and time values were formatted in R using lubridate version 1.7.10 and hms version 1.1.0 in R (Grolemund and Wickham 2011, Mueller 2021). Sensors that failed were excluded from the analysis. A small number of sensor errors were found where the temperature was outside of likely bounds (<0°C and >50°C) and so were removed.

### Sensor Position and Temperature Range

The association between temperature range, sensor position in the hive, and hive strength was tested. Observations from each sensor were summarized for each day using dplyr 1.0.7 (Wickham et al. 2021). For each sensor, days during which 285 or fewer observations were recorded (out of a possible maximum of 288 per day) were excluded from the model.

A linear mixed-effect model, as shown in Equation 2, was fitted using lme4 1.1-27.1 in R (Bates et al. 2015), estimating temperature range ($T_{(range)ijkl}$for i=1,…,Nwhere Nis the number of observations, for j=1,…,Mwhere Mis the number of hive groups, for k=1,…,Nwhere Nis the number of hives, and for l=1,…,Owhere Ois the number of days), given the horizontal distance of the sensor from the center of the hive ($cDist{}_{i}$) in combination with hive strength ($NOF_{i}$). Day-to-day variation in temperature range ($v_{l}$), variation between groups ($u_{j}$), and variation between hives within groups ($b_{k}$), were treated as random effects. The interaction between colonies with and without bees and sensor distance effects was included in the model, allowing for the relationship between temperature range and sensor distance from hive center to vary between active and inactive colonies. Significance tests for each fixed-effect variable were performed using lmerTest 3.1-3 (Kuznetsova et al. 2017).


$$\begin{array}{ll} T_{(range)ijkl}= & \;\beta_{0}+\;\beta_{1}NOF_{i}+\beta_{2}Colony_{i}+\;\beta_{3\;}cDist{\;}_{i}\\ & +\beta_{4\;}{\left(Colony\cdot cDist\right)}_{i}+u_{j}\;\;\;+\;\;\;b_{k}+v_{l}\;+\varepsilon_{ijkl} \end{array}$$ (2)

### Time to Peak Temperature

The association between colony strength and time from midnight to maximum temperature on the following day was tested using only sensors from within the hive. Observations were grouped and filtered as for the sensor position and temperature range model, and daily temperature ranges (i.e., the difference between the maximum and minimum temperatures observed by each sensor on each day) were calculated. For each sensor, the daily peak temperature was recorded and time to peak temperature from midnight prior was calculated in seconds. A linear mixed-effect model, shown in Equation 3, was fit to the data, and assessed the ability of Number of Frames ($NOF_{i}$), sensor placement distance from the center of the hive ($cDist_{i}$), and presence or absence of live bees ($Colony_{i}$), to predict time to peak temperature ($time_{\left(\;peak\right)ijkl}$for i,  j,  k,and las in Equation 2). Daily fluctuations in time to peak temperature ($v_{l}$), variation between groups ($u_{j}$), and variation between hives within groups ($b_{k}$) were treated as random effects in the model.


$$\begin{array}{ll} time_{\left(\;peak\right)ijkl}=\; & \beta_{0}+\;\beta_{1}NOF_{i}+\beta_{2}Colony_{i}+\;\beta_{3\;}cDist_{i}\\ & \;+\beta_{4\;}{\left(Colony\cdot cDist\right)}_{i}+u_{j}\;\;\;+\;\;\;b_{k}+v_{l}\;+\varepsilon_{ijkl} \end{array}$$ (3)

### Temperature Modeling

A linear mixed-effect model, as shown in Equation 4, was used to compare Number of Frames ($NOF_{i}$) with hive temperature ($T_{ijk}$for i,  j,and kas in previous equations). Fixed effects included harmonic sine and cosine curves fitted with periods of one day, half a day, and a quarter of a day, as well as time ($time_{i}$), the presence of live bees ($colony_{i}$), and sensor distance from the center of the hive ($cDist_{i}$). Variation between groups ($u_{j}$), and variation between hives within groups ($b_{k}$), were designated as random effects. The sine and cosine waves with periods of half a day were chosen to fit daily cycles in temperature, while the shorter harmonic waves in the Fourier series were selected to accommodate other observed diurnal temperature patterns, including those potentially driven by bee activity.


$$\begin{array}{ll} T_{ijk}= & \beta_{0}+\;\beta_{1}time_{i}+\beta_{2}NOF_{i}+\beta_{3}Colony_{i}+\\ & \beta_{4}sin\left(2\pi *time_{i}\right)+\beta_{5}\cos\left(2\pi *time_{i}\right)\;+\\ & \beta_{6}\sin\left(4\pi *time_{i}\right)\;+\;\beta_{7}\cos\left(4\pi *time_{i}\right)+\;\beta_{8}\sin\left(8\pi *time_{i}\right)\\ & +\;\beta_{9}\cos\left(8\pi *time_{i}\right)+\;\beta_{10}cDist_{i}+\;\;\;u_{j}\;\;\;+\;\;\;b_{k}+\;\varepsilon_{ijk} \end{array}$$ (4)

Data points from sensor 3 in Hive 8, which had a cluster count of 2, were plotted against the estimated temperature to illustrate the performance of the model. The R packages ggeffects 1.1.1 (Lüdecke 2018) and viridisLite 0.4.0 (Garnier et al. 2021) were used to predict and plot the estimated temperature change over time at sensor 3 of a hive with a cluster count of 2 as an illustration.

### Modeling with Baseline Correction

We attempted to improve model performance through baseline adjustment of the temperature measurements against ambient temperatures. Temperature data were aggregated into hourly means indexed by hive (*h*) and sensor (*s*) ($T_{hs}$). A mean hourly ambient temperature ($T_{ambient}$) was calculated from all ambient temperature sensors that functioned for the duration of the experiment (*n* = 5 sensors). Equation 4 was modified to use$T_{\left(corrected\right)hs}$, defined as $T_{hs}$–$T_{ambient}$, instead ofT, and association of hive strength with baseline adjusted temperature was determined using this linear mixed model.

### Determining Optimal Sensor Placement

Data from the four sensor positions were modeled independently to determine which placement produced a model that best predicts colony strength. Four linear mixed models were constructed in ade4 with the same random effect structure as Equation 3. The fixed effects were altered, as distance from the hive center was omitted from the model, and each model retained data from only one of the four sensor positions in the array. Akaike Information Criterion (AIC) scores were used to compare the models. AIC increases with model complexity and is reduced in models with better fit. By holding model design constant and varying the sensor used, the AIC can be used to determine the model, and thus the sensor position, that most appropriately describes colony strength (Akaike 1974).

## Results

### Hives and Hive Audits

The Number of Frames measure was used as per the Australian apiculture industry standard (Somerville and Frost 2018) i.e., without conversion to Frames of Bees (Nasr et al. 1990). Auditor assessments of cluster count (Number of Frames) were highly correlated at R = 0.919, *P* < 2.2 x 10^–16^ using Pearson’s product-moment correlation. Mean colony strength (as Number of Frames) varied from strong (7 frames) to very weak (1.1667) across the 36 hives with bees for which all sensing data was available (see below).

The full audit dataset is available on Queensland University of Technology’s Research Data Finder Repository (Cook et al. 2021b).

### Sensor and Data Logger Performance

Of the 42 sensor arrays fitted in hives, 4 arrays failed during the experiment due to short circuit or disconnection errors caused by the connector pairs, resulting in 38 hives actively recording data (*n* = 38). Two external temperature sensors were removed because they were inactive for most days of the experiment.

Of the total of 856,800 observations expected (288 observations per sensor per day, including external temperature sensors), 776,872 observations were recorded from hives, and 25,554 observations were recorded from ambient temperature sensors. Of these, 740,696 hive observations and 25,246 ambient observations remained after temperatures outside the range of 0–50°C were removed.

Missing data were observed in blocks of minutes to hours at a time, usually occurring in all sensors belonging to a particular data logger unit. Sensor arrays with high amounts of missing data were excluded from range and time to peak models to prevent distortion of the model output.

The full sensor output dataset is available on Queensland University of Technology’s Research Data Finder Repository (Cook et al. 2021b).

### Temperature Range

The median ambient temperature was 13.8°C, while the median temperature was 14.8°C for hives without bees, and 22.2°C for hives with bees. The lowest ambient temperature recorded was 1.75°C, with a maximum of 38.8°C recorded only on day one, and first and third quartiles were 9.75°C and 21.5°C respectively.

The lowest temperature recorded in a hive without bees was 1.5°C, the highest was 35.8°C, and first and third quartiles were 10.5°C and 22°C respectively. The lowest temperature recorded in a hive with bees was 8.25°C, the highest was 38°C, and first and third quartiles were 19.1°C and 26.8°C respectively.

The narrowest range of ambient temperatures recorded in any day was 14.5°C and the widest range was 32°C. Daily temperature ranges for hives without bees were between 12.5°C and 27°C, while daily temperature ranges for hives with bees were between 3.25°C and 25.5°C.

There was a significant effect of bees on within-hive temperature range regardless of colony strength. Temperature ranges were an estimated 7.39°C greater on average in hives without bees than in hives containing live bees of any colony strength (*P* = 1.01 x 10^–7^).

**Fig. 4.**
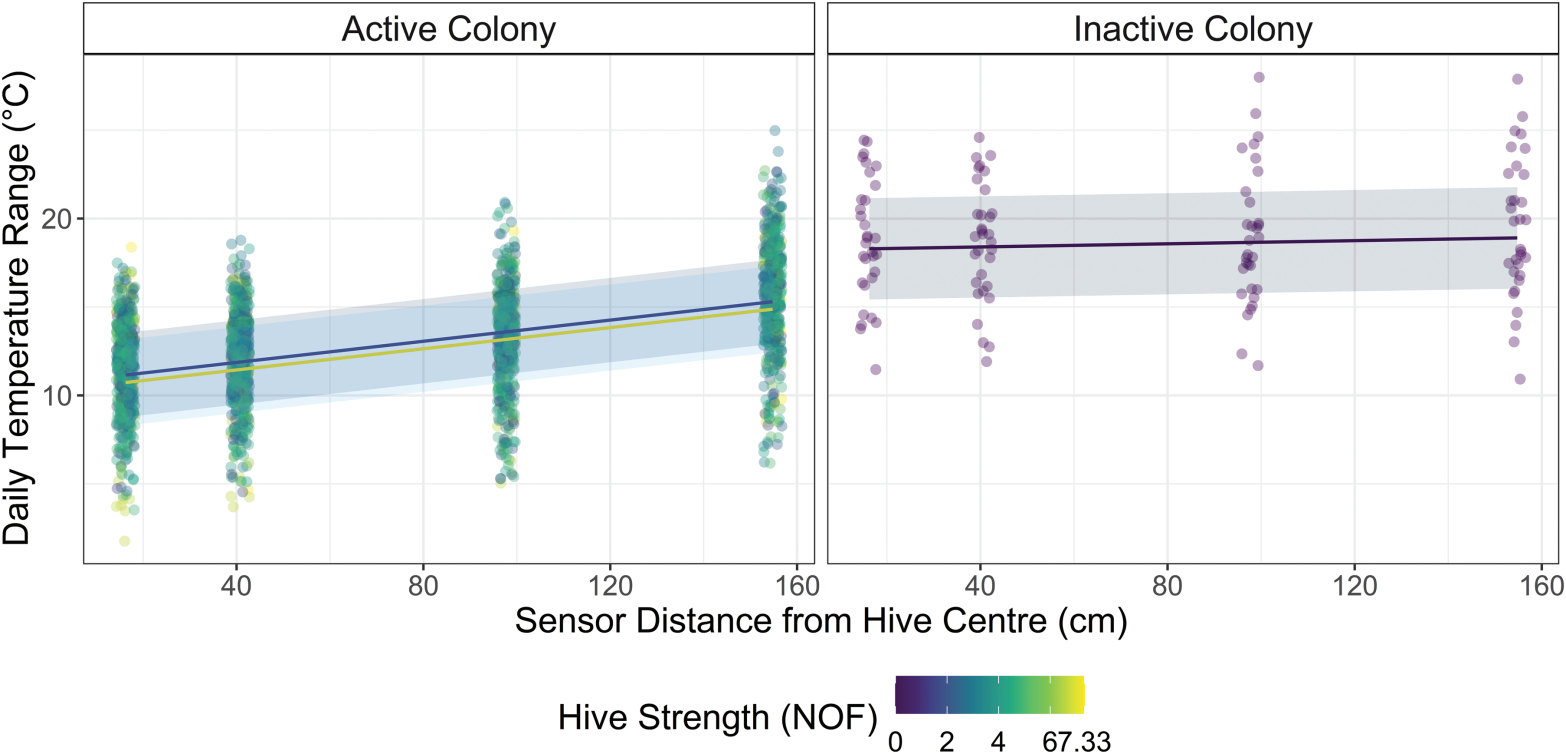
Temperature range is shown against sensor placement from the center of the hive in hives with bees (active colonies, left) and hives without bees (inactive colonies, right). Shade indicating colony strength. Predicted temperature ranges (linear) are plotted for hives with colonies of a strength of 1.166NOF (dark/blue) and 7NOF (light/yellow), and on the right in hives without bees. The presence of an active bee colony (left) significantly reduced the temperature range measured in the hive compared to those measured in hives without bees (right). Daily temperature range increases significantly with distance from the hive center in all hives, but by less in hives without bees. However, the effect of colony strength (Number of Frames) on observed temperature range is not significant (See online version for color figures).

There was a significant difference in recorded temperature range across the 4 sensor placement positions in hives with bees after controlling for random effects (Fig. 4). In hives with active bee colonies, each centimeter from the hive center resulted in an average 0.03°C increase in observed temperature range (*P* < 2 x 10^–16^). In hives with no bees, this slope was reduced by 0.025°C/cm (*P* < 2 x 10^–16^) to 0.005°C/cm. Number of Frames was not significantly associated with temperature range (*P* = 0.55).

Day-to-day variance (6.25°C^2^) and variance between hives within each group of 6 (1.59°C^2^), but not variance between groups (0.1254°C^2^), were greater than residual variance (0.90°C^2^). This suggests that daily temperature fluctuations and differences between hives were substantial sources of variability in temperature range while the grouping of hives on stands was not.

The full model is provided in the Supp Appendix A (online only).

### Time to Peak Temperature

Sensor recordings were summarized daily and 394 out of 2,659 data points were excluded due to high levels of sensor dropout. There was a significant effect from the presence of bees on the time to reach the maximum daytime temperature from midnight prior. Hives without bees reach peak temperature an average of 3,988 s (~66 min) earlier than hives with bees, regardless of colony strength (*P* = 6.24 x 10^–6^).

Neither sensor distance from the hive center (*P* = 0.130) nor colony strength (*P* = 0.633) was significantly associated with time to peak temperature within the hive after controlling for random effects, with or without bees. The full model is provided in Supp Appendix B (online only).

Unsurprisingly, day-to-day variation in time to peak temperature in seconds (5,358,070 s^2^) was higher than residual variation (3,930,420 s^2^), suggesting that variation in temperature between days is a major source of variation affecting time to peak temperature.

The full model is provided in the Supp Appendix B (online only).

### Temperature Modeling

A linear mixed model was constructed for temperature with the variables, bees (presence or absence), Number of Frames, time, sensor distance (cm center of hive), and multiple harmonic sine and cosine waves incorporated. All sine and cosine curves were significant terms in the model (*P* < 2 x 10^–16^).

The effect of presence of bees was significant. Hives without active bee colonies resulted in an average temperature of 5.55°C lower than that for hives with bees (*P* = 3.87 x 10^–5^). Sensor placement was also significant: increased distance from the center of the hive results in an average reduction in temperature of –0.016°C/cm (*P* < 2 x 10^–16^).

Importantly, the effect of Number of Frames on modeled hive temperature was significant. A one unit increase in Number of Frames was associated with an average increase of 0.36°C (*P* = 0.027).

**Fig. 5.**
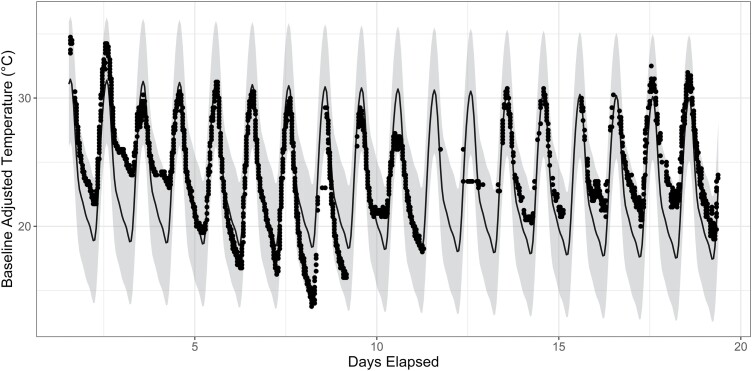
Example of observed data fit to model predictions of temperature. Temperature data from sensor 3 in hive eight (Number of Frames = 2), overlayed on the predicted temperature for sensor 3 in a hive where Number of Frames = 2. Observed datapoints largely fall within the 95% prediction interval.

Fig. 5 illustrates this by plotting the diurnal pattern of temperature data over the duration of the experiment for sensor 3 in hive 8 (Number of Frames = 2) over the model predicted temperature for a sensor at position 3 in a hive where Number of Frames = 2. The observed data points (black) largely fall within the 95% prediction interval (grey shaded).

The full model is provided in the Supp Appendix C (online only).

### Modeling with Baseline Correction

The model remained consistent with previous results for presence of bees, sensor placement, and Number of Frames after applying a baseline correction to the hive temperature data. All sine and cosine curve terms remained significant (*P* < 2 x 10^–16^).

Hives without active bee colonies were again associated with an average decrease in temperature of 5.57°C (*P* = 3.6 x 10^–5^). Sensor placement again resulted in an average decrease in temperature of 0.016 °C for each centimeter of distance from the center of the hive (*P* = 2 x 10^–16^).

In the baseline corrected model, the association between temperature and Number of Frames was the same as in the uncorrected model but was slightly less significant. A unit increase in Number of Frames corresponded to an average 0.36°C for both the corrected and uncorrected models, but with a *P* value of 0.029 in the corrected data model compared to p= 0.027 for the uncorrected data model.

The full model is provided in the Supp Appendix D (online only).

### Determining Optimal Sensor Placement

The models constructed using sensors at positions 0, 1, 2, and 3 had AICs of 59,338.17, 60,732.08, 61,953.79, and 62,897.55, respectively. Data from sensor 0, located closest to the internal wall of the hive, produced the model with the lowest AIC, however, strength was not significantly correlated with temperature in sensors 0 or 1. The full models are provided in the Supp Appendix E (online only).

## Discussion

Auditor assessments were highly correlated, demonstrating an effective audit methodology for both experienced and novice auditors. The cluster count (Number of Frames) audit method was an effective measurement of bee colony strength, enhanced using photographs and an image grading table to provide additional rigor in review of the audit count and resolution of disparity between auditors.

Hive strength requirements for almond pollination are understood to be equivalent to 8 Frames of Bees (Somerville and Frost 2018). Care must be taken to accurately define the unit of measurement of colony strength as either Number of Frames or Frames of Bees in audits and contract pollination standards. Number of Frames cannot be used in comparison to pollination standards in Frames of Bees without conversion (Nasr et al. 1990). Furthermore, the assessment of pollination efficacy and its link to colony strength requires redefinition through research, including factors such as the current audit practices and hive configurations.

The effects of bees on temperature in the hive were apparent in all the analyses of temperature data, consistent with the known activity of bees in maintaining brood (and therefore a portion of the hive) temperature in a narrow range around 35°C (Tautz 2008). Not surprisingly, hives containing bees maintained the temperature inside the hive within a much narrower range, 12.5 °C to 27 °C, compared to hives without live bees in which the range was 7.4 °C greater. In the linear mixed models, hives without active bee colonies also had a predicted model temperature 5.6°C lower than that predicted for a hive with bees in both analyses (with and without baseline correction for external temperature).

The detection of presence of bee colonies using temperature sensors has potential applications in apiaries both to detect “colony collapse”, in low-cost swarm traps (unmonitored boxes used to catch swarming colonies), and in sentinel hives used at Australian ports to detect the invasive asian honey bee (*Apis cerana* Fabricius, 1793 [Hymenoptera: Apidae]) (Beeaware.org 2021).

There was also a significant effect of presence of bees on the time between midnight and the peak daytime temperature within the hive, with the presence of bees apparently slowing the rate of hive warming. Hives without live bees reach peak temperature over an hour (66.5 min) earlier than hives with bees, regardless of colony strength. This “thermal lag” might result from active heating and cooling through thermal regulation by bees, through the movement of bees in and out of the hive during foraging, and a greater thermal mass of bees, honey, and brood within the hive (Tautz 2008, Mitchell 2016, Cook et al. 2021a).

The variation in the time to peak temperature at different sensor points across the hive was not significant, suggesting that distribution of brood, honey, pollen, and wax have little impact on rate of heating and cooling despite their known contribution to thermal mass (Cook et al. 2021a). However, the presence of bees significantly modified the range of temperature at points across the hive: sensors recorded an increased range of temperatures over a 24-h period in sensors closer to the wall edge of the hive compared to the middle of the hive when bees were present. This is consistent with the known activity of bees in maintaining a narrow range of temperature close to 35°C around the developing brood, which is typically located towards the center of the hive, and effects of thermal mass (Kleinhenz et al. 2003, Humphrey and Dykes 2008, Tautz 2008, Stabentheiner et al. 2010, Cook et al. 2021a).

We may also speculate that the hot, endothermic foragers returning at dusk may be occupying the peripheries of the hive, as seen in Humphrey and Dykes (2008) study, resulting in increased positive temperature range. Furthermore, thermal management such as heat shielding and thermal relocation from the brood area to the extremities of the nest may be used to mitigate brood overheating and may also be responsible for this increased temperature range at the edge of the hive (Starks and Gilley 1999, Bonoan et al. 2014).

Simple measures of temperature range and rate of warming (time to peak temperature) did not correlate significantly with Number of Frames. However, Number of Frames was significantly correlated with predicted temperature by incorporating multiple harmonic sine and cosine terms in both the linear mixed models (with and without baseline correction for ambient temperature) once random factors had been excluded. These terms capture diurnal temperature cycles within the hive in the model. It is therefore important that algorithms associated with temperature sensing technology used to determine colony strength should include factors that accommodate diurnal temperature cycles.

The modeling showed a sensitive and significant response with a one unit increase in Number of Frames associated with a 0.36°C increase in temperature. Thus, algorithms using temperature sensor data can be used to estimate colony strength. However, this study was conducted within a range of cooler ambient temperatures that suggest the colonies would be more involved in colony heating behaviors, and thus requires repetition in additional climates and ambient temperatures to determine the actions of cooling behaviors on the model.

A substantial proportion of variance in time to peak temperature and temperature range was caused by day-to-day variation. This variation, likely the effect of fluctuating temperature and weather patterns, may obscure the variation driven by the activity of bees in live monitoring applications using single sensor technology, necessitating the use of paired external sensors to correct for fluctuations.

Sensor placement was significantly correlated with greater temperature range in hives, and more strongly in hives with bees (0.03°C/cm from hive center), but sensor placement was not significantly correlated with time to peak temperature. Sensor placement was also significant in both the linear mixed models that incorporated diurnal temperature cycles, detecting a predicted decrease in temperature of 0.016°C per cm increased distance from the center of the hive.

The AIC values associated with the models using data from sensors at positions 0 and 1, closer to the edge of the hive, indicated that these models best fit the data, but colony strength was not a significant predictor of temperature in these models. The models based on sensors 2 and 3 had a higher AIC value (i.e., did not fit the data as well) but in these models temperature was significantly correlated with Number of Frames.

Previous published studies have explored the placement of sensors at the center of frames 5 and 6 in center of the brood box, between last frame and brood box wall, on top of frames 5 and 6, and one on top of central frames in the honey super (Meikel et al. 2015). Most commercial sensing applications suggest placing sensors over the more thermally stable center point. The results in this paper test and validate the relevance of this central, top placement in the context of lateral placement of sensors on top of frames within a hive.

This is useful information for research and commercial applications: Again, the underpinning biological explanation for this may be a result of the greater capacity of stronger colonies to manage hive temperature in the central cluster or brood area. However, it must be noted that the brood cluster is not always in a central location and may move laterally or vertically to favor the thermal profile of the hive, environment, or season, affecting optimal sensor positioning (Owens 1971, Mitchell 2016).

These results support several potential applications of thermal sensing in practice, firstly, in the use of temperature sensors to detect an absence of bees using simple parameters (temperature range and time to peak temperature) as well as using more complex linear mixed models. More significantly, the linear mixed modeling incorporating diurnal thermal cycles support the use of temperature sensing not only to detect presence or absence of bees, but also to report changes in colony strength.

Many apiarists move hives over long distances for both pollination services and seasonal honey production. The results support the use of temperature sensors with upload capability to alert apiarists to events such as colony collapse, severe disease, or absconding (or swarming) in distant locations through changes in colony strength. Furthermore, the results validate the use of single sensors placed in the geometric center of the hive over brood (Stalidzans and Berzonis 2013, Zacepins et al. 2016, Meikle et al. 2015, 2017).

The linear mixed model results support the use of temperature sensing as a potential method to determine colony strength, but with limitations. These experiments were conducted in an environment where the ambient temperature was below optimal brood nest temperature (~35°C). It is expected that the thermal behavior of the bee colony will change as ambient temperature approaches 35°C, as occurs in Australian summers, when the colony may be oscillating between heating and cooling activities to balance endothermic forager impacts, colony thermal metabolic output, and ambient temperature change. Above 35°C, cooling mechanisms are expected to predominate (Bonoan et al. 2014, Tautz 2008, Bordier et al. 2017).

We suggest that this short term, preliminary study be repeated with more rigorous, objective audits that quantify food stores, brood area, and their locations within the hive, and measure the bee population, allowing correlation of colony temperature profiles to a complete bee colony model. We also suggest that further studies be made into the correlation of colony strength and temperature in alternate environments, in differing seasons, and in conditions where colony cooling behavior is dominant, as well as the “cross over conditions” between heating and cooling behaviors.

The study identified important discrepancies between standardized audit methods and industry standards used in practice by pollination services, which require further research and revision. However, the significant correlation between colony strength determined by the industry-standard Number of Frames method used in this study and results from temperature sensing suggesting that temperature sensing could have relevance in improving hive auditing.

In conclusion, this work identifies parameters for the use of temperature sensors to estimate presence and absence of bees and colony strength, and validated the conventional placement of sensors in the geometric center of the hive. It validates the use of temperature sensing combined with statistical models that include patterns of diurnal thermal cycles to refine and improve the practical use of temperature sensing in apiculture for pollination services.

## Supplementary Material

toac034_suppl_Supplementary_Appendices
