# Supplementary material for: Temperature Sensing and Honey Bee Colony Strength
Source: J Econ Entomol. 2022 May 6;115(3):715–23. doi: 10.1093/jee/toac034 (PMC9175291; doi:10.1093/jee/toac034)
Supplement: toac034_suppl_Supplementary_Appendices [file toac034_suppl_supplementary_appendices.docx]

## Appendix A: Sensor position and temperature range model.

REML criterion at convergence: 6312.5

Scaled residuals:

Min 1Q Median 3Q Max

-6.3728 -0.5798 -0.0373 0.5392 5.5438

Random effects:

Groups Name Variance Std.Dev.

Hive:Group(Intercept) 1.5925 1.2619

Day (Intercept) 6.2486 2.4997

Group (Intercept) 0.1254 0.3541

Residual 0.9014 0.9494

Number of obs: 2197, groups: Hive:Unit, 38; Day, 17; Unit, 7

Fixed effects:

Estimate Std. Error df t value Pr(>|t|)

(Intercept) 1.082e+01 8.702e-01 4.401e+01 12.433 5.37e-16 ***

Strength -8.888e-02 1.469e-01 3.427e+01 -0.605 0.549

cDist 2.993e-02 3.893e-04 2.141e+03 76.875 < 2e-16 ***

ColonyInactive Colony 7.393e+00 1.102e+00 3.432e+01 6.707 1.01e-07 ***

cDist:ColonyInactive Colony -2.548e-02 1.614e-03 2.141e+03 -15.786 < 2e-16 ***

## Appendix B: Time to peak temperature model.

REML criterion at convergence: 39724

Scaled residuals:

Min 1Q Median 3Q Max

-4.5023 -0.5769 -0.0492 0.5450 7.2197

Random effects:

Groups Name Variance Std.Dev.

Hive:Group(Intercept) 695462 833.9

Day (Intercept) 5358070 2314.8

Group (Intercept) 0 0.0

Residual 3930420 1982.5

Number of obs: 2197, groups: Hive:Unit, 38; Day, 17; Unit, 7

Fixed effects:

Estimate Std. Error df t value Pr(>|t|)

(Intercept) 50165.563 701.507 33.986 71.511 < 2e-16 ***

Strength 48.330 100.247 36.699 0.482 0.633

cDist -1.196 0.789 2142.579 -1.515 0.130

ColonyInactive Colony -3938.494 742.041 35.161 -5.308 6.24e-06 ***

## Appendix C: Time series model

REML criterion at convergence: 3438119

Scaled residuals:

Min 1Q Median 3Q Max

-4.8050 -0.6197 -0.0063 0.6182 6.0514

Random effects:

Groups Name Variance Std.Dev.

Hive:Group(Intercept) 1.929e+00 1.389e+00

Group (Intercept) 3.402e-09 5.833e-05

Residual 6.070e+00 2.464e+00

Number of obs: 740696, groups: Hive:Unit, 38; Unit, 7

Fixed effects:

Estimate Std. Error df t value Pr(>|t|)

(Intercept) 2.382e+01 6.519e-01 3.502e+01 36.543 < 2e-16 ***

Time -8.479e-02 5.569e-04 7.407e+05 -152.256 < 2e-16 ***

Strength 3.630e-01 1.574e-01 3.500e+01 2.306 0.0272 *

ColonyInactive Colony -5.548e+00 1.179e+00 3.502e+01 -4.707 3.87e-05 ***

sin((2 * pi) * Time) -3.199e+00 4.034e-03 7.407e+05 -792.951 < 2e-16 ***

cos((2 * pi) * Time) -4.592e+00 4.070e-03 7.407e+05 -1128.313 < 2e-16 ***

sin((4 * pi) * Time) 1.063e+00 4.047e-03 7.407e+05 262.656 < 2e-16 ***

cos((4 * pi) * Time) 1.678e+00 4.053e-03 7.407e+05 414.153 < 2e-16 ***

sin((8 * pi) * Time) -1.675e-01 4.049e-03 7.407e+05 -41.371 < 2e-16 ***

cos((8 * pi) * Time) -2.704e-01 4.049e-03 7.407e+05 -66.799 < 2e-16 ***

cDist -1.591e-02 5.342e-05 7.407e+05 -297.858 < 2e-16 ***

## Appendix D: Time series model with baseline temperature comparison.

REML criterion at convergence: 272916.5

Scaled residuals:

Min 1Q Median 3Q Max

-5.6764 -0.6428 -0.0188 0.6292 5.7406

Random effects:

Groups Name Variance Std.Dev.

Hive:Group(Intercept) 1.926e+00 1.388e+00

Group (Intercept) 3.329e-11 5.770e-06

Residual 4.373e+00 2.091e+00

Number of obs: 63202, groups: Hive:Unit, 38; Unit, 7

Fixed effects:

Estimate Std. Error df t value Pr(>|t|)

(Intercept) 6.268e+00 6.521e-01 3.507e+01 9.612 2.31e-11 ***

Strength 3.580e-01 1.574e-01 3.500e+01 2.274 0.0292 *

cDist -1.589e-02 1.552e-04 6.316e+04 -102.371 < 2e-16 ***

Time 1.182e-01 1.612e-03 6.316e+04 73.350 < 2e-16 ***

ColonyInactive Colony -5.577e+00 1.179e+00 3.500e+01 -4.732 3.60e-05 ***

sin((2 * pi) * Time) -2.179e+00 1.173e-02 6.316e+04 -185.704 < 2e-16 ***

cos((2 * pi) * Time) 3.203e+00 1.181e-02 6.316e+04 271.165 < 2e-16 ***

sin((4 * pi) * Time) 1.310e+00 1.175e-02 6.316e+04 111.559 < 2e-16 ***

cos((4 * pi) * Time) -8.737e-01 1.179e-02 6.316e+04 -74.131 < 2e-16 ***

sin((8 * pi) * Time) -9.525e-01 1.178e-02 6.316e+04 -80.873 < 2e-16 ***

cos((8 * pi) * Time) 1.056e+00 1.175e-02 6.316e+04 89.862 < 2e-16 ***

## Appendix E: Models with different sensors.

### Sensor 0

REML criterion at convergence: 59314.2

Scaled residuals:

Min 1Q Median 3Q Max

-4.8168 -0.6421 0.0316 0.6924 3.9785

Random effects:

Groups Name Variance Std.Dev.

Hive:Group(Intercept) 0.8762 0.9361

Group (Intercept) 0.0000 0.0000

Residual 3.0474 1.7457

Number of obs: 14954, groups: Hive:Unit, 36; Unit, 7

Fixed effects:

Estimate Std. Error df t value Pr(>|t|)

(Intercept) 4.509e+00 4.421e-01 3.431e+01 10.20 6.35e-12 ***

Strength 1.865e-01 1.066e-01 3.401e+01 1.75 0.0892 .

Time 9.900e-02 2.766e-03 1.491e+04 35.80 < 2e-16 ***

sin((2 * pi) * Time) -2.357e+00 2.014e-02 1.491e+04 -117.04 < 2e-16 ***

cos((2 * pi) * Time) 2.346e+00 2.027e-02 1.491e+04 115.71 < 2e-16 ***

sin((4 * pi) * Time) 1.444e+00 2.015e-02 1.491e+04 71.66 < 2e-16 ***

cos((4 * pi) * Time) -4.154e-01 2.023e-02 1.491e+04 -20.53 < 2e-16 ***

sin((8 * pi) * Time) -8.662e-01 2.021e-02 1.491e+04 -42.85 < 2e-16 ***

cos((8 * pi) * Time) 9.889e-01 2.017e-02 1.491e+04 49.04 < 2e-16 ***

### Sensor 1

REML criterion at convergence: 60708.1

Scaled residuals:

Min 1Q Median 3Q Max

-5.2707 -0.6440 0.0190 0.6775 4.9165

Random effects:

Groups Name Variance Std.Dev.

Hive:Group(Intercept) 1.757e+00 1.325390

Group (Intercept) 3.461e-08 0.000186

Residual 3.372e+00 1.836229

Number of obs: 14920, groups: Hive:Unit, 36; Unit, 7

Fixed effects:

Estimate Std. Error df t value Pr(>|t|)

(Intercept) 4.799e+00 6.242e-01 3.417e+01 7.688 5.94e-09 ***

Strength 3.572e-01 1.506e-01 3.401e+01 2.372 0.0235 *

Time 1.225e-01 2.913e-03 1.488e+04 42.054 < 2e-16 ***

sin((2 * pi) * Time) -2.268e+00 2.120e-02 1.488e+04 -106.975 < 2e-16 ***

cos((2 * pi) * Time) 3.362e+00 2.135e-02 1.488e+04 157.441 < 2e-16 ***

sin((4 * pi) * Time) 1.374e+00 2.123e-02 1.488e+04 64.702 < 2e-16 ***

cos((4 * pi) * Time) -9.192e-01 2.130e-02 1.488e+04 -43.159 < 2e-16 ***

sin((8 * pi) * Time) -9.419e-01 2.128e-02 1.488e+04 -44.254 < 2e-16 ***

cos((8 * pi) * Time) 1.071e+00 2.124e-02 1.488e+04 50.444 < 2e-16 ***

### Sensor 2

REML criterion at convergence: 61929.8

Scaled residuals:

Min 1Q Median 3Q Max

-5.8566 -0.6369 0.0221 0.6689 4.2351

Random effects:

Groups Name Variance Std.Dev.

Hive:Group(Intercept) 2.680 1.637

Group (Intercept) 0.000 0.000

Residual 3.622 1.903

Number of obs: 14954, groups: Hive:Unit, 36; Unit, 7

Fixed effects:

Estimate Std. Error df t value Pr(>|t|)

(Intercept) 5.240e+00 7.702e-01 3.412e+01 6.804 7.8e-08 ***

Strength 4.343e-01 1.859e-01 3.401e+01 2.337 0.0255 *

Time 1.357e-01 3.016e-03 1.491e+04 45.011 < 2e-16 ***

sin((2 * pi) * Time) -2.096e+00 2.195e-02 1.491e+04 -95.463 < 2e-16 ***

cos((2 * pi) * Time) 3.827e+00 2.211e-02 1.491e+04 173.131 < 2e-16 ***

sin((4 * pi) * Time) 1.291e+00 2.198e-02 1.491e+04 58.709 < 2e-16 ***

cos((4 * pi) * Time) -1.159e+00 2.205e-02 1.491e+04 -52.540 < 2e-16 ***

sin((8 * pi) * Time) -9.818e-01 2.204e-02 1.491e+04 -44.547 < 2e-16 ***

cos((8 * pi) * Time) 1.113e+00 2.199e-02 1.491e+04 50.626 < 2e-16 ***

### Sensor 3

REML criterion at convergence: 62873.5

Scaled residuals:

Min 1Q Median 3Q Max

-5.1315 -0.6297 0.0151 0.6548 4.9711

Random effects:

Groups Name Variance Std.Dev.

Hive:Group(Intercept) 3.682 1.919

Froup (Intercept) 0.000 0.000

Residual 3.869 1.967

Number of obs: 14942, groups: Hive:Unit, 36; Unit, 7

Fixed effects:

Estimate Std. Error df t value Pr(>|t|)

(Intercept) 5.319e+00 9.023e-01 3.410e+01 5.895 1.17e-06 ***

Strength 4.534e-01 2.178e-01 3.401e+01 2.082 0.0449 *

Time 1.423e-01 3.118e-03 1.490e+04 45.631 < 2e-16 ***

sin((2 * pi) * Time) -1.999e+00 2.270e-02 1.490e+04 -88.063 < 2e-16 ***

cos((2 * pi) * Time) 4.020e+00 2.285e-02 1.490e+04 175.887 < 2e-16 ***

sin((4 * pi) * Time) 1.218e+00 2.272e-02 1.490e+04 53.597 < 2e-16 ***

cos((4 * pi) * Time) -1.243e+00 2.280e-02 1.490e+04 -54.485 < 2e-16 ***

sin((8 * pi) * Time) -9.954e-01 2.279e-02 1.490e+04 -43.679 < 2e-16 ***

cos((8 * pi) * Time) 1.127e+00 2.273e-02 1.490e+04 49.572 < 2e-16 ***
